# Factors Showing the Growing Relation Between Vitamin D, Metabolic Syndrome, and Obesity in the Adult Population: A Systematic Review

**DOI:** 10.7759/cureus.27335

**Published:** 2022-07-27

**Authors:** Humaira Kauser, Jaimee J Palakeel, Mazin Ali, Phani Chaduvula, Sanika Chhabra, Smriti Lamsal Lamichhane, Vaiishnavi Ramesh, Collins O Opara, Farhana Y Khan, Gargi Kabiraj, Lubna Mohammed

**Affiliations:** 1 Medicine, California Institute of Behavioral Neurosciences & Psychology, Fairfield, USA; 2 Neurology, California Institute of Behavioral Neurosciences & Psychology, Fairfield, USA; 3 Medicine, Michigan State University, Grand Rapids, USA; 4 Family Medicine, California Institute of Behavioral Neurosciences & Psychology, Fairfield, USA; 5 Radiation Medicine, California Institute of Behavioral Neurosciences & Psychology, Fairfield, USA; 6 Pathology, California Institute of Behavioral Neurosciences & Psychology, Fairfield, USA; 7 Medicine, Mata Gujri Memorial Medical College & Lions Seva Kendra Hospital, Bolpur, IND; 8 Internal Medicine, California Institute of Behavioral Neurosciences & Psychology, Fairfield, USA

**Keywords:** 25-hydroxyvitamin d, dysmetabolic syndrome, low vitamin d, obesity, bmi, insulin resistance, syndrome x, metabolic syndrome, hypovitaminosis d, vitamin d deficiency

## Abstract

Several theories suggest an inverse association between increasing adiposity, particularly abdominal fat, and low vitamin D levels. As a result, several routes are likely to impact how vitamin D, obesity, and metabolic syndrome (MetS) interact. This systematic study followed Preferred Reporting Items for Systematic Reviews and Meta-Analyses (PRISMA) standards. A comprehensive PubMed, PubMed Central, Google Scholar, and ScienceDirect database search was conducted for published papers over the previous five years. Studies were identified using the following criteria 1) participants, interventions, and outcomes (PIO) components, 2) free full text, 3) studies published in English, and 4) human studies, including systematic and narrative reviews and cross-sectional, observational studies, were among the inclusion and exclusion criteria. In total, 151 articles were returned, and 16 duplicates were rejected. After verifying the titles and abstracts of these records using the review's PIO components and eligibility criteria, 17 received a 70% or above score. On review of the literature, the release of adiponectin from fatty tissues was inversely correlated with body weight and BMI suggesting a link between vitamin D deficiency and insulin resistance.

## Introduction and background

Vitamin D deficiency is a global health problem affecting approximately one billion individuals worldwide [1]. Vitamin D is primarily required to absorb calcium, phosphate, and magnesium in the gut and prevent rickets. However, most of it is produced in the skin by UV irradiation of 7-dehydrocholesterol. The active form of vitamin D, 1,25-dihydroxy vitamin D3 (1,25(OH)2D3), regulates transcriptional activity and is necessary for calcium homeostasis and metabolism. Vitamin D also has functions in adipogenesis, glucose-insulin homeostasis, cell growth, non-alcoholic fatty liver disease, diabetes, insulin resistance, and metabolic syndrome [2,3]. Table 1 shows the vitamin D ranges [4].

**Table 1:** Ranges of vitamin D according to the CDC Source: CDC [4]

Category of vitamin D status	Serum 25OHD levels
At risk of vitamin D deficiency	Serum 25OHD < 30nmol/L (12ng/ml)
At risk of vitamin D inadequacy	Serum 25OHD 30-49nmol/L (12-19ng/ml)
Sufficient in vitamin D	Serum 25OHD 50-125nmol/L (20-50ng/ml)
Possibly harmful vitamin D	Serum 25OHD >125nmol/L (50ng/ml)

Metabolic syndrome (MetS) is a collection of cardiovascular risk disorders that, when present together, increase the risk of diabetes and heart disease [5]. The number of persons affected with MetS has risen considerably in the previous two decades, now accounting for 20% to 25% of all adults in the United States and posing a significant public health threat [6]. MetS is defined by the presence of central (intraabdominal) obesity, hypertension (HT), hyperglycemia, raised triglyceride and cholesterol levels in the blood, along with low high-density lipoproteins (HDLs) and insulin resistance (IR). However, the classification and management of MetS is still up for debate. MetS is linked to an increased risk of cardiovascular disease and type 2 diabetes and is one of the most thoroughly studied factors [7].

Obesity has been more common worldwide in the previous 30 years, along with an increase in type 2 diabetes and vitamin D deficiency. According to the World Obesity Federation, if current trends continue, over one billion persons will be obese, and 2.7 billion will be overweight by 2025 [8]. Obesity is described as a build-up of abnormal or excessive fat and body weight of more than 20% of the ideal weight. Food addiction, genetic mutation, inadequate physical activity, endocrine disease, and poor dietary status contribute to the build-up of body fat [9]. Table 2 shows BMI ranges [10].

**Table 2:** BMI ranges according to the CDC Source: CDC [10]

BMI ranges	Category of weight
BMI <18.5	Underweight range
BMI 18.5 to 24.9	Normal or healthy weight range
BMI 25.0 to 29.9	Overweight range
BMI >30.0	Obese range

In epidemiological studies, vitamin D levels in the blood have shown to be inversely related to MetS. Vitamin D is known to help the absorption of calcium and phosphorus in the gut. Vitamin D deficiency lowers intracellular calcium levels, which inhibits insulin release by cells, further worsening glucose tolerance. According to another study, vitamin D increases insulin receptors, which are necessary for glucose metabolism and insulin responsiveness [11].

According to increasing data, vitamin D insufficiency may be a risk factor for MetS. In some studies, increased vitamin D levels have been shown to lower the risk of MetS development. Supplementing with vitamin D, for example, has been shown to help obese and overweight patients lose abdominal visceral fat tissue. In addition, a three-month double-blind, randomized clinical trial of vitamin D treatment in women demonstrated that it reduced body fat mass regardless of BMI. These results support the theory that vitamin D levels are negatively related to MetS prevalence [11].

On the other hand, other research studies have been unable to replicate this association among these specific groups of people. As a result, investigations on the negative relationship between blood 25 hydroxyvitamin D (25(OH)D) and MetS were inconsistent. Furthermore, in the middle-aged and older population, there is a lack of sex-based data associating blood 25(OH)D levels with MetS. [11] In a cross-sectional study of Australians aged 18-75 years, the prevalence of MetS was 65 percent lower in the upper quartile of serum vitamin D concentrations than in the lowest quartile [1].

There are various theories for the inverse link between increased adiposity, particularly abdominal obesity, and low plasma Vitamin D levels. Still, none of them has been able to explain the relationship altogether. As a result, many different potential mechanisms affect how vitamin D, obesity, and MetS interact. Consequently, additional study is required to uncover the causative relationship and to discover and establish vitamin D supplementation as a therapy for obesity and MetS [9].

However, investigations on the clinical implications of vitamin D have yielded mixed results, which raises the following question. Is vitamin D insufficiency in people with obesity just a coincidence, or does it significantly impact the development and progression of obesity and MetS [9]? For the reasons stated above, this study's primary goal (aim) is to perform a comprehensive literature evaluation to ascertain the function of vitamin D in developing metabolic syndrome and obesity and its influence on the adult population.

## Review

Methods

PRISMA 2020 standards were used to perform this systematic review [12].

Eligibility Criteria

Participants, interventions, and outcomes (PIO) components were used to select the studies. The studies selected included randomized controlled trials, observational studies, systematic reviews, and meta-analyses. In addition, the inclusion and exclusion criteria were added- papers from the last five years, free full text written in English, and papers with human subjects only.

Databases and Search Strategy

The PubMed, PubMed Central (PMC), Google Scholar, and Science Direct databases were used to perform a comprehensive search. All databases were searched from April 2, 2022, to April 20, 2022. The field search employed in the procedure was chosen based on keywords found in prior research and Medical Subject Headings (Mesh), as shown in Table 3, depending on the databases used.

**Table 3:** The search strategy in the databases with their corresponding filters

Databases	Keywords	Search strategy	Filters	Search results
PubMed	Vitamin D Deficiency OR hypovitaminosis D OR low vitamin D OR low levels of serum 25-hydroxyvitamin D OR low sunshine vitamin AND Metabolic syndrome OR Syndrome X OR insulin resistance syndrome OR dysmetabolic syndrome OR abdominal obesity metabolic syndrome AND Obesity OR elevated BMI OR high BMI>30 OR overweight OR increase abdominal circumference	Vitamin D Deficiency OR hypovitaminosis D OR low vitamin D OR low levels of serum 25-hydroxyvitamin D OR low sunshine vitamin OR ( "Vitamin D Deficiency/adverse effects"[Majr] OR "Vitamin D Deficiency/blood"[Majr] OR "Vitamin D Deficiency/chemistry"[Majr] OR "Vitamin D Deficiency/classification"[Majr] OR "Vitamin D Deficiency/diet therapy"[Majr] OR "Vitamin D Deficiency/drug therapy"[Majr] OR "Vitamin D Deficiency/epidemiology"[Majr] OR "Vitamin D Deficiency/history"[Majr] OR "Vitamin D Deficiency/metabolism"[Majr] OR "Vitamin D Deficiency/mortality"[Majr] OR "Vitamin D Deficiency/physiology"[Majr] OR "Vitamin D Deficiency/physiopathology"[Majr] OR "Vitamin D Deficiency/psychology"[Majr] OR "Vitamin D Deficiency/therapeutic use"[Majr] OR "Vitamin D Deficiency/therapy"[Majr] ) AND Metabolic syndrome OR Syndrome X OR insulin resistance syndrome OR dysmetabolic syndrome OR abdominal obesity metabolic syndrome OR ( "Metabolic Syndrome/classification"[Majr] OR "Metabolic Syndrome/complications"[Majr] OR "Metabolic Syndrome/diagnosis"[Majr] OR "Metabolic Syndrome/diet therapy"[Majr] OR "Metabolic Syndrome/drug therapy"[Majr] OR "Metabolic Syndrome/epidemiology"[Majr] OR "Metabolic Syndrome/mortality"[Majr] OR "Metabolic Syndrome/pathology"[Majr] OR "Metabolic Syndrome/physiology"[Majr] OR "Metabolic Syndrome/physiopathology"[Majr] OR "Metabolic Syndrome/statistics and numerical data"[Majr] ) AND Obesity OR elevated BMI OR high BMI>30 OR overweight OR increase abdominal circumference OR ( "Obesity/classification"[Majr] OR "Obesity/complications"[Majr] OR "Obesity/diagnosis"[Majr] OR "Obesity/diet therapy"[Majr] OR "Obesity/drug therapy"[Majr] OR "Obesity/history"[Majr] OR "Obesity/metabolism"[Majr] OR "Obesity/pathology"[Majr] OR "Obesity/physiology"[Majr] OR "Obesity/physiopathology"[Majr] OR "Obesity/statistics and numerical data"[Majr] ) - 52,340	Last five years, free full-text full text, English and Humans only	44
PubMed Central	Vitamin D and Metabolic Syndrome and Obesity	Vitamin D and Metabolic syndrome and Obesity - 9309	Last five years, open access only	53
Google Scholar	Vitamin D and Metabolic Syndrome and Obesity	Vitamin D and Metabolic syndrome and Obesity - 17,200	2017- 2022 review articles only	47
Science Direct	Vitamin D and Metabolic Syndrome and Obesity	Vitamin D and Metabolic syndrome and Obesity - 1419	2017-2022, review articles and research articles, open access only	7

All references were gathered in Microsoft Excel 2021 (Microsoft, Redmond, Washington) to eliminate duplicates. The data was first vetted based on titles and abstracts, with irrelevant studies being eliminated. The full-text papers were retrieved after the review.

Risk of Bias

The remaining full articles were assessed for quality and risk of bias using the following tools: Newcastle-Ottawa Scale (NOS), Assessment of Multiple Systematic Reviews 2 (AMSTAR 2), Scale for the Assessment of Narrative Review Articles 2 (SANRA 2), Appraisal tool for Cross-Sectional Studies (AXIS). Each tool has its own set of criteria and grading system. When a tool receives a "YES," "PARTIAL YES," or "1," it is awarded a point. When the number "2" appears, it is worth two points. Each evaluation instrument required a minimum score of 70%. Table 4 shows the quality appraisal tools of the articles.

**Table 4:** Quality assessment of each type of study SANRA 2 - Scale for the Assessment of Narrative Review Articles; AMSTAR 2 - Assessment of Multiple Systematic Reviews 2; AXIS - Appraisal Tool for Cross-sectional Studies; NOS - Newcastle-Ottawa Scale; RCTs - randomized controlled trials; RoB - risk of bias

Quality assessment tool	Type of study	Items and their characteristics	Total score	Accepted score (70%)	Accepted studies
SANRA 2 (25)	Narrative reviews	Six items: Justification of the article's value to the readership, Goals or question formulation, literature search described, referencing, scientific reason, and appropriate presentation of data. Scored as 0, 1, or 2.	12	9	Eight: Goncalves et al. [13]; Contreras-Bolívar et al. [14]; Wimalawansa et al. [15]; Melguizo-Rodríguez et al. [3]; Hyppönen et al. [16]; Rocha dos Santos et al. [17]; Sacerdote et al. [18]; Kheiri et al. [2]
AMSTAR 2 (9)	Systematic reviews and Meta-analyses	Sixteen items: (1) Did the review's research questions, and inclusion criteria incorporate PICO components? (2) Is there an explicit indication in the review report indicating the review techniques were defined prior to the review's execution, and does the report justify any substantial deviations from the protocol? (3) Did the review authors explain why they chose the research designs they did for the review? (4) Was a comprehensive literature search technique used by the review authors? (5) Did the review writers choose the studies in duplicate? (6) Was data extraction duplicated by the review authors? (7) Did the review authors give a list of papers that were eliminated and explain why? (8) Were the included studies adequately described by the review authors? (9) Was there a solid approach used by the review authors to assess the risk of bias (RoB) in the individual studies included in the review? (10) Are the funding sources for the research included in the review reported by the review authors? (11) If meta-analysis was justified, did the review authors apply adequate statistical procedures to combine the results? (12) If a meta-analysis was conducted, did the authors consider the influence of RoB in individual studies on the meta-analysis or other evidence synthesis results? (13) When interpreting/discussing the review's findings, did the authors consider RoB in individual studies? (14) Is there an acceptable explanation and discussion of any heterogeneity in the review results provided by the review authors? (15) Did the review authors do an appropriate analysis of publication bias (small study bias) and address its anticipated influence on the review's outcomes if they used quantitative synthesis? (16) Did the authors disclose any possible conflicts of interest, such as any funds they received to perform the review? Scored as YES or NO. Partial Yes was considered as a point.	16	12	Four: AlAnouti et al. [6]; Aquina et al. [19]; Lu Yu et al. [20]; Rafiq et al. [8]
AXIS (7)	Cross-sectional studies	Twenty items: (1) Were the aims/objectives of the study clear? (2) Was the research design suitable for the stated goal? (3) Was the sample size justified? (4) Was the target/reference population clearly defined? (5) Was the sample frame drawn from a suitable population basis to ensure that it accurately reflected the target/reference population under investigation? (6) Was the selection process likely to select subjects/participants representing the target population under investigation? (7) Were measures undertaken to address and categorize non-responders? (8) Were the risk factors and outcome variables measured per the study's objectives? (9) Were the risk factor and outcome variables accurately quantified with instruments/measurements that have previously been trialed, piloted, or published? (10) Is it clear what was used to determine statistical significance and/or precision estimates? (11) Were the methods well-described such that they could be replicated? (12) Were the primary data adequately described? (13) Does the response rate concern non-response bias? (14) Was there any mention of non-responders if it was relevant? (15) Were the results internally consistent? (16) Were the findings for all of the analyses mentioned in the procedures presented? (17) Were the author's discussions and conclusions justified by the results? (18) Have the study's shortcomings been discussed? (19) Was there any funding sources of conflicts of interest that may affect the author's interpretation of the results? (20) Was ethical approval or consent of participants attained?	20	14	Five: Mutt et al. [21]; Kaseb et al. [22]; Ghadieh et al. [23]; Liu et al. [24]; Weldegiorgis et al. [11]
NOS (3)	Case-control and cohort studies	Eight items: (1) The exposed cohort's representativeness (2) Selection of the non-exposed cohort (3) Determination of exposure (4) Evidence indicating the desired outcome did not exist at the start of the research (5) Cohort comparability based on design or analysis* (6). Assessment of outcome (7) Was the follow-up period long enough for results to occur? (8) Adequacy of follow-up of cohorts scoring was done by placing a point on each category. Scored as 0, 1, 2. * Maximum of two points are allotted in this category.	8	6	0

Data Collection, Items, and Analysis

Due to the apparent inter-variability of studies, such as the heterogeneity of participants, treatments, and clinical outcomes, this systematic review evaluates these experiments and reviews based on their results, applicability, and limitations. Narrative reviews, observational studies, cross-sectional studies, and systematic reviews were all read, evaluated, and tabulated. The first author's year, research type, illness, inclusion and exclusion criteria, significant findings, and funding sources were all collected from each study.

Outcome Assessment

Data collection, selection, appraisal, and analysis were all made by two independent investigators at each stage. If the group could not agree on whether or not an article was eligible, the complete text was evaluated.

Table 5 shows a brief overview of the studies used as references for this systematic review.

**Table 5:** Overview of studies used as references MetS - metabolic syndrome

Authors	Year of publication	Conclusion/outcome
Lips et al. [25]	October 2017	Fasting plasma glucose levels were shown to be somewhat lower, or insulin resistance improved in investigations. These effects are more seen in people who have vitamin D insufficiency and poor glucose tolerance.
Goncalves et al. [13]	October 2017	Fat-soluble micronutrients such as vitamin D could contribute to preventing MetS due to their central role as hormone regulators.
Kheiri et al. [2]	June 2018	Vitamin D deficiency is linked to hypertension and increased cardiovascular and all-cause mortality.
Rafiq et al. [8]	August 2018	Low vitamin D levels are linked to increased BMI in both diabetic and non-diabetic patients in studies.
Vranić et al. [9]	August 2018	The primary therapy option for both obesity-related dysmetabolic conditions and vitamin D insufficiency should be to change one's lifestyle through a balanced diet and exercise.
Hyppönen et al. [16]	September 2018	Vitamin D has a variety of physiological and biochemical actions that may help to offset the negative effects of obesity on metabolism and minimize the risk of metabolic irregularities and tissue damage caused by obesity.
Park et al. [7]	December 2018	Low blood vitamin D levels have been linked to obesity, diabetes, insulin resistance, and cardiovascular disorders, including hypertension.
Mutt et al. [21]	May 2019	Low vitamin D levels and other lifestyle variables such as food choices and physical inactivity are risk factors for MetS in older people in the Northern Hemisphere (65°North).
Weldegiorgis et al. [11]	June 2019	A lower serum 25(OH)D level in middle-aged males is strongly linked to MetS.
Greco et al. [26]	June 2019	The close association between obesity, glucose homeostasis, and hypovitaminosis D is due to obesity-related inadequate sun exposure and outdoor activities. Vitamin D storage in adipose tissue (lipophilic properties), insulin secretion, sensitivity, and the immune system also shows an association.
Miao et al. [27]	February 2020	Vitamin D pills appear to be a viable strategy to lessen the impact of these disorders. Finally, vitamin D supplementation may provide a new foundation for medical treatment.
AlAnouti et al. [6]	October 2020	Before reaching any firm conclusions on vitamin D level and its therapeutic relevance for dyslipidemia in MetS, more research is needed.
Melguizo-Rodríguez et al. [3]	February 2021	Plasma vitamin D concentrations have been found to have an inverse connection with the characteristics of MetS, such as increased glucose, total cholesterol, low-density lipoprotein, lipids, glycated hemoglobin, and a high BMI.
Jha et al. [5]	April 2021	Vitamin D plays a function in more than bone health and bone growth. Thus, an appropriate amount should be consumed.
Lee et al. [1]	June 2021	In cohort and cross-sectional studies, a 25-nmol/L increase in blood vitamin D content was related to 15%, and 20% decreased risks of MetS, respectively, according to a dose-response meta-analysis.

Results

Study Selection and Quality Assessment

**Figure 1:**
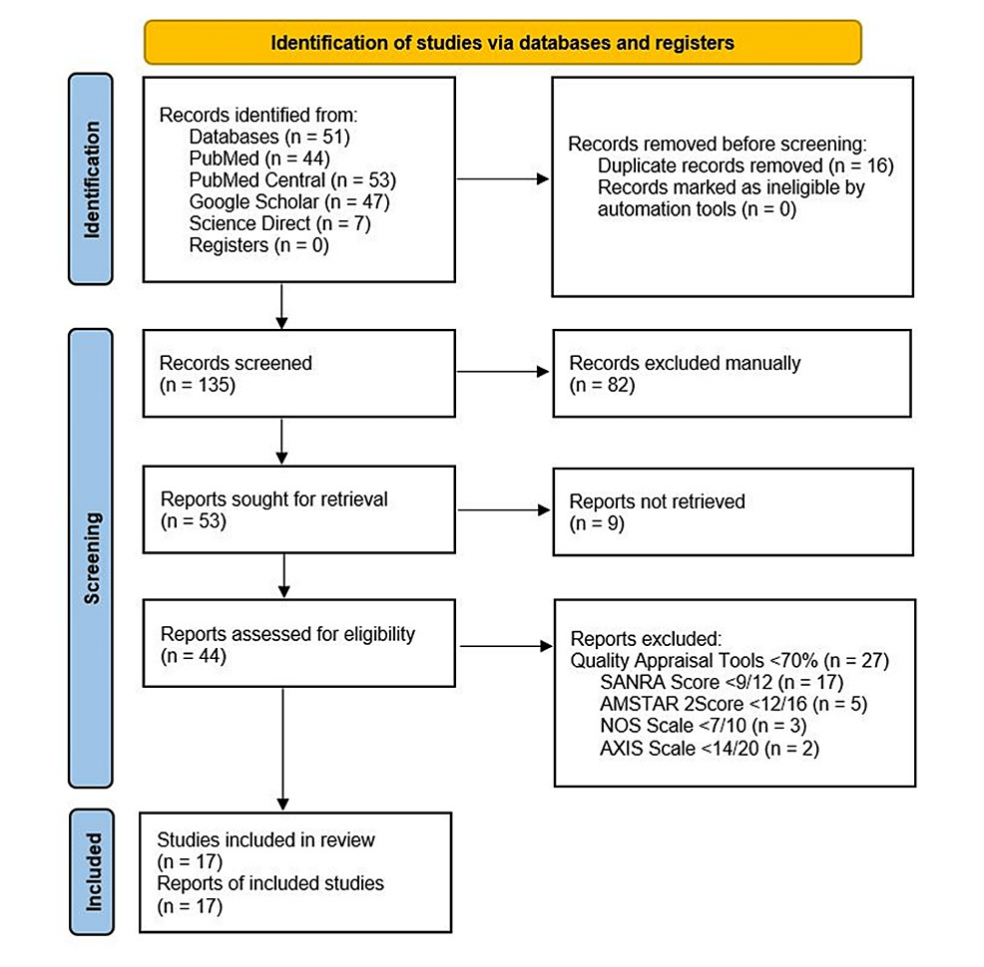
Flow chart of the study search selection SANRA - Scale for the Assessment of Narrative Review Articles; AMSTAR 2 - Assessment of Multiple Systematic Reviews 2; NOS - Newcastle Ottawa Scale; AXIS - Appraisal Tool for Cross-sectional studies

There were 151 potentially relevant articles in the database search, out of which 16 duplicates were removed. After that, we had 135 articles left when the titles and abstracts of these records were screened based on the review's PIO elements and eligibility criteria. After the initial screening, 82 papers were excluded manually. Of the remaining 53 records, nine were not accessible. Finally, a quality assessment for 44 articles was done, and 17 studies with a score of 70% and above were accepted in the review. There were no new resources. The graphic below is a PRISMA flow diagram depicting the screening and study selection process (Figure 1).

Discussion

This section will discuss the pathophysiology of vitamin D and metabolic syndrome, how metabolic syndrome, vitamin D, and obesity are interlinked, and studies showing the relationship between MetS, vitamin D, and obesity.

Vitamin D Pathophysiology

**Figure 2:**
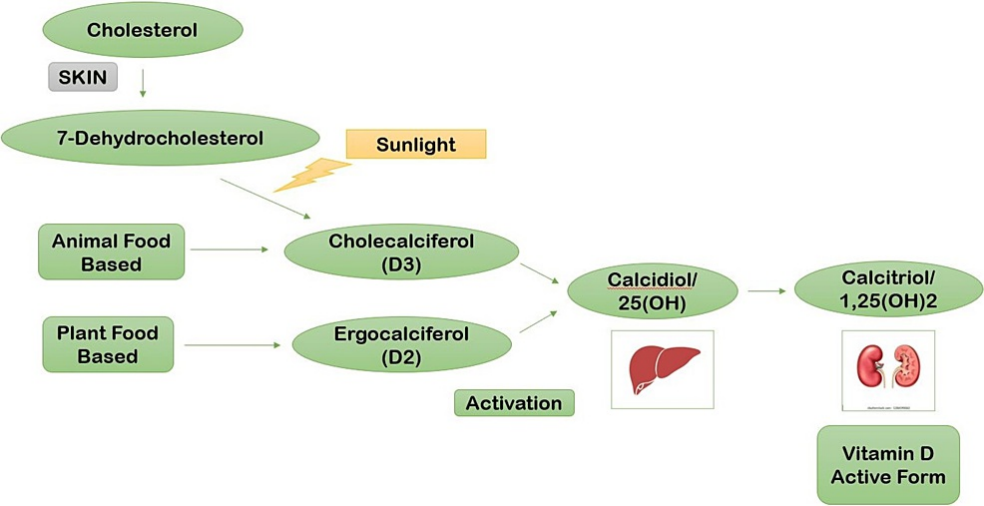
Pathophysiology of vitamin D Inspired by Melguizo-Rodríguez et al. [3]

Vitamin D is the most important fat-soluble vitamin that is involved in calcium and phosphorus metabolism as well as skeletal homeostasis. Cholecalciferol, or vitamin D3, is the most common form of vitamin D, which is made from 7-dehydrocholesterol, a precursor to cholesterol. Animal (cholecalciferol) and vegetable (ergocalciferol) diets also contain it. Vitamin D takes two hydroxylations in the body to become physiologically active, the first in the liver and the second in the kidney, resulting in the 1,25(OH)2 vitamin D or calcitriol form [3]. Figure 2 shows the pathophysiology of vitamin D.

Metabolic Syndrome Pathophysiology

**Figure 3:**
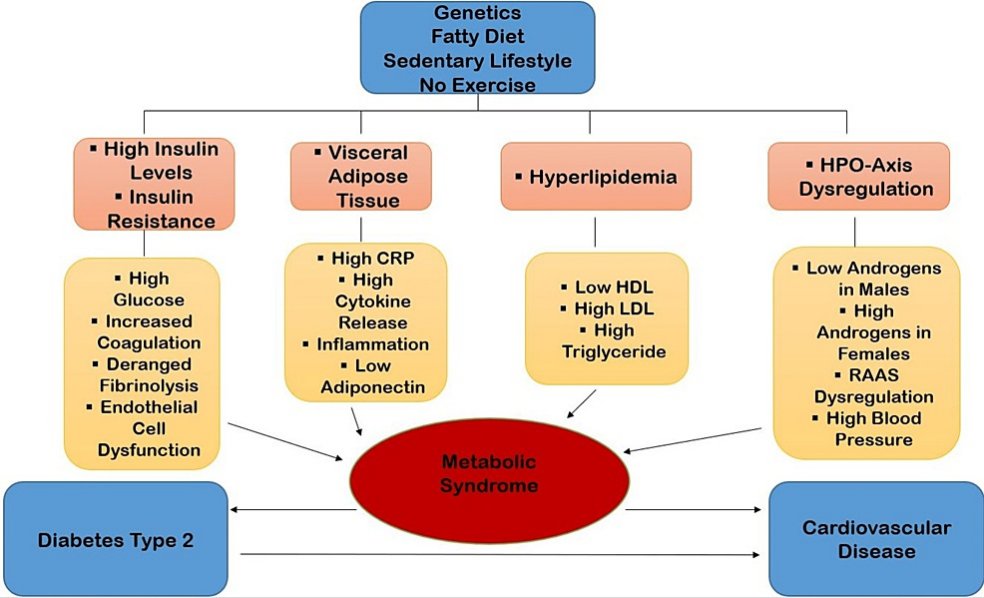
Pathophysiology of metabolic syndrome HPO - hypothalamic-pituitary-ovarian; CRP: C-reactive protein; HDL - high-density lipoprotein; LDL - low-density lipoprotein; RAAS: renin-angiotensin-aldosterone system Created by author Humaira Kauser

Genetics, a fatty diet, and a sedentary lifestyle are all variables that contribute to metabolic syndrome. As a result, increased insulin levels, visceral fatty tissue deposits, raised lipid levels, and HPO (hypothalamic-pituitary axis) dysregulation occur. Finally, it plays a role in the development of diabetes and heart disease. Figure 3 shows the pathophysiology of metabolic syndrome.

Vitamin D Deficiency and Obesity

Obesity is a pathological condition defined by an abnormally high body weight due to the build-up of fat mass caused by gene mutations, a sedentary lifestyle, endocrine abnormalities, or poor nutritional status. Obesity is a significant global health issue linked to comorbidities such as metabolic syndrome, diabetes, hypertension, lung illnesses, non-alcoholic fatty liver disease, kidney problems, heart disease, skeletal abnormalities, and cancer. Furthermore, low blood vitamins such as vitamin B1 (thiamine), folate, and vitamin D are frequently associated with obesity [26].

Several epidemiological and experimental studies have demonstrated an inverse association between circulating vitamin D levels, central adiposity, and the development of obesity, as well as an association between low levels of serum 25-hydroxyvitamin D (25(OH)D), obesity, and metabolic syndrome, including both children and adults, and regardless of age. Furthermore, research has found that the link between obesity and the lack of vitamin D is not dependent on vitamin D supplementation and dietary changes. Excess adiposity, rather than insufficient intake, increases the risk of vitamin D deficiency in overweight and obese adults. However, the relationship between low vitamin D levels and various factors such as body fat, sun exposure, food intake, and exercise has yet to be discovered. Inadequate sun exposure and outdoor activities and changed expression of the vitamin D receptor (VDR) in adipose tissue are likely to be the main causes of the link between obesity and vitamin D insufficiency in obese people [26].

The genomic responses to vitamin D are caused by stereospecific interactions between the active metabolite (1,25(OH)2D) and its internal VDR, a steroid hormone receptor. After attaching to the hormone, VDR produces a heterodimer with the retinoid-X receptor (RXR) that binds to particular vitamin D response elements (VDREs) on DNA sequences, causing some gene products to be expressed or trans-repressed. Because it is produced by pancreatic β-cells, adipose tissue, and skeletal muscle, VDR regulates the expression of numerous genes that govern calcium/phosphate balance, cellular proliferation and differentiation, immunological response, glucose tolerance, and insulin sensitivity [26].

Adipogenesis is a series of processes that cause preadipocytes to develop into mature adipocytes, as well as adipocyte hypertrophy, which leads to obesity. In vitro research performed 30 years ago revealed that vitamin D might inhibit adipogenesis and that triglyceride accumulation in 3T3-L1 preadipocytes exposed to 1,25(OH)2D was decreased by 50% when compared to cells that were not treated. Following the findings, it was discovered that Cyp27b1, the gene encoding the enzyme that converts 25(OH)D to 1,25(OH)2D, is expressed in rodent and human fat tissue and that vitamin D inhibits adipogenesis via a competitive mechanism involving peroxisome proliferator-activated receptor-gamma (PPAR) and VDR. VDR and PPAR share a common heterodimeric binding partner, RXR, and higher VDR expression is linked to a decrease in PPAR-induced adipogenesis and a decrease in the mitotic clonal adipocyte population. Another study on murine mesenchymal cells treated with vitamin D confirmed reduced differentiation of adipocytes from bone marrow-derived cells [26].

VDR is also linked to thermogenesis regulation since it regulates uncoupling protein 1 (UCP1) and protein 2 (UCP2). The nuclear VDR activation of 1,25(OH)2D dramatically reduces the expression of UCP2 in human fat cells, which appears to play a role in the pathogenetic pathways of insulin resistance and diabetes development. Furthermore, in a clinical investigation, a low-energy diet and vitamin D treatment reduced fat mass and body weight. In contrast, vitamin D administration prevented diet-induced obesity in an in vivo mouse model [26].

Vitamin D is a fat-soluble vitamin, and body fat mass serves as a storehouse for vitamin D. Vitamin D may build up in the body and be extensively distributed. However, it is primarily stored in adipose tissue before being gradually released. The larger pool of the fat tissue in the visceral and subcutaneous areas impounds vitamin D and its compounds in obese people, lowering its bioavailability. An inverse relationship of 25(OH)D to body mass and adiposity was observed in obese persons following bariatric surgery [26].

Finally, obese people have decreased levels of solar exposure due to a lack of aerobic exercise, mobility, and psychological discomfort [26].

Vitamin D Deficiency, Insulin Resistance, and Diabetes

Insulin resistance, the most prevalent cause of type 2 diabetes mellitus (T2DM), is caused by insulin signaling pathway dysregulation, which is caused by reduced insulin sensitivity. Obesity and hypovitaminosis D is associated in people and experimental animal models, according to emerging evidence. Although the underlying molecular pathways are unknown, vitamin D deficiency has been linked to insulin resistance and T2DM [26].

Vitamin D affects glycemic homeostasis through various processes, which include glucose-mediated insulin synthesis/secretion by -cells, regulation of hepatic and peripheral glucose uptake, and regulation of inflammation [26].

VDR and the 1-hydroxylase enzyme are present in pancreatic β-cells, and 1,25(OH)2D has been shown to act on pancreatic cells overwhelmed by inflammation and vitamin D deficiency. Furthermore, appropriate vitamin D levels are required to maintain extracellular calcium levels outside the cells and calcium inflow into -cells for insulin secretion. VDR signaling may play a key role in glucose-induced insulin secretion [26].

VDR signaling enhances insulin-induced glucose uptake in the liver, adipose, and muscle fiber tissues, and 1,25(OH)2D directly causes the insulin receptor activation and protein in both humans and experimental animal models, along with regulating insulin production and secretion. In addition, 1,25(OH)2D has been shown to increase GLUT-4 expression in muscle cells and promote its translocation in animal study adipocytes in vivo [26].

Low-grade chronic inflammation, which is present more in obese persons due to increased pro-inflammatory cytokine production by macrophages and adipocytes, is another important factor in obesity and insulin resistance affecting the immune system. Multiple studies have shown that in the presence of abdominal adiposity, vitamin D deficiency is linked to inflammation and decreased insulin sensitivity, whereas vitamin D treatment improves both outcomes. Vitamin D reduces adipocyte chemokine and cytokine release, as well as monocyte chemotaxis. Its effects on the systemic and tissue-specific inflammatory response have been attributed to a variety of factors, including suppression of the NF-κβ pathway, T-helper cell anti-inflammatory activation, reduction of toll-like receptor 4 (TLR-4) expression (which reduces the differentiation of dendritic cells) [26].

**Figure 4:**
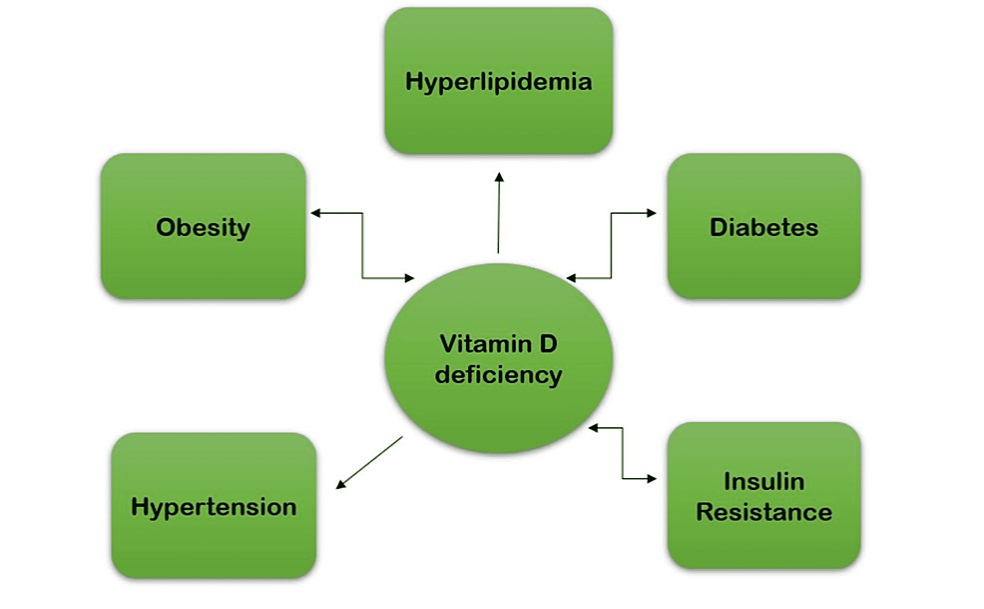
Interlinkage between metabolic syndrome, vitamin D, and obesity Inspired by Vranić et al. [9]

Adiponectin, which is secreted by fatty tissues in an opposite ratio to body weight and body mass index, could represent a potential link between vitamin D deficiency and insulin resistance. Adiponectin has been shown to have insulin-sensitizing capabilities in tissues as well as modulatory effects on gluconeogenesis, and its sensors are found in pancreatic β-cells. It is not clear how vitamin D interacts with adiponectin because adiponectin and glucose metabolism are mediated by osteocalcin, an osteoblast-derived protein that is impacted by vitamin D. 1,25(OH)2D influences adipogenesis through a VDR-dependent process that interacts with PPAR, they are most likely connected [26]. Figure 4 shows the interlinkage between metabolic syndrome, vitamin D, and obesity.

Vitamin D levels and Components of MetS

Of the 319 MetS participants, 26.3% were vitamin D deficient, whereas 55.2% were insufficient. Low vitamin D levels were powerfully linked to waist circumference, with 25.8% of the individuals deficient and 55.6% inadequate (p=0.001). The participants with low vitamin D levels had greater triglyceride (TG), high-density lipoprotein (HDL), and blood pressure (BP) than those with adequate vitamin D levels. However, the difference was not statistically significant (Table 6). The fasting blood glucose (FBG) (110 mg/dl) (p=0.058), on the other hand, demonstrated a modest but negligible link with low vitamin D levels [21]. Table 6 shows vitamin D in relation to metabolic syndrome, waist circumference, triglyceride, HDL cholesterol, blood pressure, and fasting glucose [21].

**Table 6:** 25OHD level in relation to metabolic syndrome, waist circumference, triglyceride, HDL cholesterol, blood pressure, and fasting glucose HDL - high-density lipoprotein Source: International Diabetes Federation (IDF) [21]

Components	Total cases	25OHD level						p-value	Vitamin D supplementation				p-value
		Deficiency		Insufficiency		Sufficiency			No		Yes		
		(<50 nmol/L)		(50–75 nmol/L)		(≥75 nmol/L)							
		Number	Percentage	Number	Percentage	Number	Percentage		Number	Percentage	Number	Percentage	
Metabolic syndrome (IDF)													
No	313	54	17.3	187	59.7	72	23	0.016	141	45.1	172	54.9	<0.001
Yes	319	84	26.3	176	55.2	59	18.5		183	57.4	136	42.6	
Waist circumference (cm)													
Men < 94 & Women < 80	175	20	11.4	109	62.3	46	26.3	<0.001	83	47.4	92	52.6	0.043
Men ≥ 94 & Women ≥ 80	457	118	25.8	254	55.6	85	18.6		241	52.7	216	47.3	
Triglyceride (mg/dL)													
<150	374	80	21.4	222	59.4	72	19.2	0.491	183	48.9	191	51.1	0.074
≥150	259	58	22.4	144	55.6	57	22		144	55.6	115	44.4	
HDL cholesterol (mg/dL)													
Men ≥ 40 & women ≥ 50	394	85	21.6	232	58.9	77	19.5	0.732	193	49	201	51	0.034
Men < 40 & women<50	239	53	22.2	134	56.1	53	21.7		134	56.1	105	43.9	
Blood pressure (mmHg)													
No (<130/85)	106	19	17.9	58	54.8	29	27.3	0.192	43	40.6	63	59.4	0.006
Yes (≥130/85)	527	120	22.8	305	57.9	102	19.3		282	53.5	245	46.5	
Fasting glucose (mg/dL)													
<100	296	59	19.9	164	55.4	73	24.7	0.058	128	43.2	168	56.8	<0.001
≥100	337	80	23.7	199	59.1	58	17.2		197	58.5	140	41.5	

The MetS and our individuals' vitamin D supplementation histories were compared. Of the 319 MetS patients, 57.4% did not use vitamin D supplements, whereas 42.6% did. Except for the TG (p = 0.07; Table 3), those who did not receive vitamin D had significant associations with one or more MetS components. As a result, vitamin D supplementation was linked to a reduced risk of MetS. We also looked at the possible causes of low vitamin D levels in the blood [21]. *Associated Studies*

A health survey of 1790 Japanese employees found a higher negative relationship between serum 25(OH)D level and MetS among those with morbidly obese than those with average weight. The model has been appropriately modified (for age, sex, smoking, alcohol consumption, physical activity, calcium intake, and BMI), and the chances of having any of the five MetS components were lower in the vitamin D sufficient group than in the vitamin D-deficient group (by 23% for high fasting plasma glucose, 13% for high triglycerides and low HDL-cholesterol, 48% for high blood pressure, and 8% for increased waist circumference); however, none of these associations were significant [13].

According to the Third National Health and Nutrition Examination Survey (NHANES III), among 6228 people, the highest quartile of 25(OH)D (81 nmol/l) protected against the development of T2DM. When this is compared to the lowest quartile (25(OH)D 43.9 nmol/l), the odds ratio for diabetes in the top quartile was 0.17 in Mexican Americans and 0.25 in non-Hispanic Whites [25].

A multivariate linear regression analysis discovered a substantial positive relationship between vitamin D insufficiency and waist circumference, HDL, and high triglyceride levels. A cross-sectional survey of 1205 Qatari people found that 28 % had MetS. 64% of this group was deficient in vitamin D, and those with MetS had a lower 25(OH)D level by 8%. Furthermore, high blood pressure was inversely related to 25(OH)D levels, especially in vitamin D deficient people [13].

A substantial negative connection between serum 25(OH)D and glycated hemoglobin (HbA1c) was discovered in the 1958 British Birth Cohort. HbA1c was lower in the group with serum 25(OH)D 25 nmol/l when compared to the group with serum 25(OH)D 75 nmol/l (5.37 percent against 5.12 percent), with an even higher difference among obese people [25].

One hundred twenty-six people with MetS and vitamin D insufficiency (serum level 20 ng/ml) were classified as obese or non-obese using a BMI threshold of 28 kg/m2. In a randomized control, the obese group had decreased blood vitamin D (p=0.05), and fasting plasma insulin, at the start. Despite a substantial rise in blood vitamin D levels in both the obese (from 11.4 to 26.8 ng/ml, p=0.05) and non-obese (from 17.4 to 38.7 ng/ml, p=0.05) groups after the one-year intervention (700 IU/day of vitamin D, or placebo), MetS risk variables did not change in treated patients [13].

Limitations

The included research was confined to the English language in only four databases from the last five years, which means that good publications from prior years may have been overlooked. Grey literature and other databases were not included. This article describes the fat-soluble vitamin D and its association with metabolic syndrome and obesity. Other fat-soluble vitamins A, E, and K are not mentioned, and their association needs to be studied in more detail. This study does not show the association of vitamin D with metabolic syndrome separately in males and females. This study is not precise about whether vitamin D supplementation helps prevents diabetes mellitus and how vitamin D affects hypertension, and if vitamin D supplementation will show changes in insulin resistance or a two-hour glucose plasma test.

## Conclusions

This systematic review thoroughly examines the elements that show a growing relationship between vitamin D, metabolic syndrome, and obesity in adults. Vitamin D insufficiency is uncommon since it may be produced at sufficient levels in the body by simply exposing oneself to sunshine for 10 minutes every day. This review aimed to explore the effects of vitamin D on adipogenesis, with a focus on illnesses associated with adipose metabolic abnormalities. Adipogenesis is influenced by vitamin D in numerous ways. VDR is expressed in adipose tissue, which produces, stores, and degrades active vitamin D. Due to the close link between obesity, glucose homeostasis, and hypovitaminosis D, having a good vitamin D status may be desirable, and based on the known mechanisms involved in the action of vitamin D. Obese individuals may represent the primary recipients of vitamin D's effects on insulin sensitivity modulation and T2DM prevention. Vitamin D might help at least one of the five cardiovascular risk factors linked to MetS, with a more substantial benefit in healthy people. Supplementing with vitamin D may be more effective in the early stages of metabolic disease. However, the results of vitamin D supplementation intervention trials to improve insulin resistance and glucose tolerance are still debatable. Then there is the need for more long-term randomized controlled studies and meta-analyses focusing on obese patients with vitamin D deficiency and the careful selection of dosage, dosing schedule, and achievement of target 25(OH)D blood levels.
